# Willingness to pay for assisted reproductive technologies among individuals with infertility in China

**DOI:** 10.1093/heapol/czaf045

**Published:** 2025-07-17

**Authors:** Chaofan Li, Hongbin Cong, Stephen Jan, Lei Si, Ling Geng, Shunping Li

**Affiliations:** Department of Social Medicine and Health Management, School of Public Health, Cheeloo College of Medicine, Shandong University, 44 Wenhuaxi Road, Jinan 250012, China; NHC Key Laboratory of Health Economics and Policy Research (Shandong University), 44 Wenhuaxi Road, Jinan 250012, China; Center for Health Management and Policy Research, Shandong University (Shandong Provincial Key New Think Tank), 44 Wenhuaxi Road, Jinan 250012, China; Center for Health Preference Research, Shandong University, 44 Wenhuaxi Road, Jinan 250012, China; State Key Laboratory of Reproductive Medicine and Offspring Health, Center for Reproductive Medicine, Institute of Women, Children and Reproductive Health, Shandong University, 44 Wenhuaxi Road, Jinan 250012, China; The George Institute for Global Health, Faculty of Medicine and Health, University of New South Wales, 300 Barangaroo Ave, Sydney, NSW 2000, Australia; The George Institute of Global Health UK, Faculty of Medicine, Imperial College London, White City Campus, London W12 0SN, United Kingdom; School of Health Sciences, Western Sydney University, 183 Narellan Road, Campbelltown, NSW 2560, Australia; Translational Health Research Institute, Western Sydney University, Locked Bag 1797, Penrith, NSW 2751, Australia; State Key Laboratory of Reproductive Medicine and Offspring Health, Center for Reproductive Medicine, Institute of Women, Children and Reproductive Health, Shandong University, 44 Wenhuaxi Road, Jinan 250012, China; Department of Social Medicine and Health Management, School of Public Health, Cheeloo College of Medicine, Shandong University, 44 Wenhuaxi Road, Jinan 250012, China; NHC Key Laboratory of Health Economics and Policy Research (Shandong University), 44 Wenhuaxi Road, Jinan 250012, China; Center for Health Management and Policy Research, Shandong University (Shandong Provincial Key New Think Tank), 44 Wenhuaxi Road, Jinan 250012, China; Center for Health Preference Research, Shandong University, 44 Wenhuaxi Road, Jinan 250012, China

**Keywords:** willingness to pay, patients, health economics

## Abstract

Infertility, a widely prevalent condition globally, incurs high economic burdens. Assisted reproductive technologies (ARTs) are effective treatments, but public health financing in low- and middle-income countries (LMICs) rarely covers ART services. In China, where birth rates are declining, willingness to pay (WTP) can inform insurance reimbursement policies by reducing out-of-pocket expenses. However, there is no consensus on WTP thresholds for assessing the cost-effectiveness of fertility treatments in LMICs. This study aimed to assess WTP for ART among individuals with infertility in China. Data were obtained from a cross-sectional survey conducted at five hospitals across different geographical and socioeconomic regions in China. Individuals with infertility were recruited using a quota sampling method. A contingent valuation method was employed, with three hypothetical WTP scenarios developed to present detailed information on the success rates, costs, and the treatment processes of *in vitro* fertilization (IVF), artificial insemination (AI), and preimplantation genetic testing (PGT). A total of 570 individuals with infertility participated in the survey [94.4% female; mean (standard deviation) age: 33.0 (4.7) years]. The sampled respondents were willing to pay renminbi (RMB) 30 163 [$4259, 95% confidence interval (CI): RMB 29 650–30 675] for IVF, RMB 6046 ($854, 95% CI: RMB 5987–6106) for AI, and RMB 47 234 ($6669, 95% CI: RMB 46 435–48 033) for PGT. These WTPs were equivalent to 0.34, 0.07, and 0.53 times the GDP per capita in China, respectively. Older age and male-factor or unexplained infertility were significantly associated with lower WTP (*P* < .05), while higher education and patient-physician communication about costs were positively associated with WTP (*P* < .05). These findings suggest that public health insurance schemes should establish appropriate cost-effectiveness thresholds and reimbursement ceilings for ART to improve affordability and access. Incorporating patient-physician communication about cost into clinical practice may facilitate shared decision-making and potentially increase patients’ perceived value of ART.

Key messages

- Infertility is widely prevalent and incurs high economic burden globally and in China.
- Assisted reproductive technologies (ARTs) are effective treatments for infertility, while they are rarely covered through public health financing in low- and middle-income countries.
- The Chinese infertility patients were willing to pay renminbi (RMB) 30 163 ($4259) for *in vitro* fertilization, RMB 6046 ($854) for artificial insemination and RMB 47 234 ($6669) for preimplantation genetic testing, respectively.
- The findings on willingness to pay for ART implied that public health insurance schemes should establish appropriate cost-effective thresholds and ceilings for ART care to enhance affordability for infertility patients and boost fertility rates in China.

## Introduction

Infertility is defined as the “failure to achieve a pregnancy after 12 months or more of regular unprotected sexual intercourse” (Vander Borght and Wyns 2018, World Health Organization 2023a). The prevalence of infertility is ∼17.5% globally and 23.2% in the Western Pacific Region (World Health Organization 2023b). Infertility not only has negative impacts on patients’ physical and mental health, but also imposes considerable financial burdens on households, frequently resulting in catastrophic health expenditures due to out-of-pocket treatment costs (World Health Organization 2023a). As infertility treatment services are a core component in promoting sexual and reproductive health and fulfilling essential human rights, the provision of available, affordable, and high-quality fertility care has been recognized as an important objective of health systems.

However, prohibitive costs remain a major barrier to accessing fertility care, particularly assisted reproductive technologies (ARTs) (Dyer *et al*. 2013, Seifer *et al.* 2018, Njagi *et al*. 2023). Several high-income countries, such as Australia and the UK, cover some of the costs of ART services through public subsidies or social health insurance schemes, significantly reducing patients’ out-of-pocket expenses for infertility treatment (Chambers *et al*. 2009, International Federation of Fertility Societies 2022). However, most low- and middle-income countries (LMICs) lack public financing mechanisms for ART. Consequently, individuals with infertility in these settings face high risks of catastrophic health expenditure (Njagi *et al*. 2023). The global rise in infertility will increase demand for ART, highlighting the need to implement targeted financial interventions to improve equitable access to fertility care (Liang *et al*. 2025). It is therefore critical for governments in LMICs to consider public funding for ART.

Willingness-to-pay (WTP), the maximum amount an individual is willing to pay for a given service or health improvement (Lin *et al*. 2013), is a widely used tool to assist healthcare policymakers in determining which health technologies should be prioritized for coverage under public health insurance schemes (Facey *et al*. 2017, Keller *et al*. 2023). It reflects the monetary value individuals place on health technologies and can inform thresholds for health technology assessment. However, a previous systematic literature review found that only limited research was performed to investigate WTP in LMICs (Fenwick *et al*. 2023 ). Among 17 empirical WTP studies, most were conducted in the USA, Australia, Israel, Brazil and European countries (Fenwick *et al*. 2023). Only one study focused on women with infertility in Hong Kong, China (Lai *et al*. 2021). Research on WTP for infertility treatment in mainland China and LMICs remains scarce. The review also found that there were no standard WTP benchmarks for ARTs, and that factors associated with WTP included treatment outcomes, age, education level, and income (Fenwick *et al*. 2023). There is an urgent need to estimate WTP thresholds for infertility treatments based on well-defined outcome measures, such as clinical pregnancy or live birth.

China, the largest LMIC in the world, has an infertility prevalence of ∼25% among couples of reproductive age (Zhou *et al*. 2018). The ARTs, including artificial insemination (AI), *in vitro* fertilization (IVF), and preimplantation genetic testing (PGT), are approved by the National Health Commission of China for the treatment of infertility (Bai *et al*. 2020, National Health Commission of the People’s Republic of China 2025). Although the availability, efficacy, and safety of ART in mainland China are comparable to those in the USA and European countries, access to ART in China remains a challenge for patients with infertility due to the exclusion of ART costs from health insurance schemes (Bai *et al*. 2020, Qiao *et al*. 2021). In China, the clinical pregnancy rates for AI, IVF, and PGT are ∼20%, 50%, and 60%, respectively (Zhang *et al*. 2022). However, the high costs of ARTs—around renminbi (RMB) 5000–8248 ($1253–2067) for AI, RMB 20 000–80 000 ($5013–20 050) for IVF, and RMB 30 000–90 000 ($7519–22 556) per cycle for PGT—are important barriers for individuals with infertility (Bai *et al*. 2018, Qiao *et al*. 2021, Wang *et al*. 2022). Over the past decade, both academic researchers and the public have advocated for ART subsidies through public funds or health insurance schemes (Wang *et al*. 2022). Since 2023, select ART procedures have been included in China’s health insurance benefit package (Global Times 2025). The government has made efforts to identify ART services with high cost-effectiveness for prioritization in health insurance coverage and to determine appropriate reimbursement rates, pricing mechanisms, and coverage ceilings.

In this context, assessing WTP for ART among individuals with infertility is crucial to inform health insurance reimbursement decisions. Given the absence of established WTP for ART in China, this study aims to estimate WTP values and identify their determinants, thereby addressing a critical evidence gap to inform future policymaking and health technology assessment.

## Materials and methods

### Data source

The data used in this study were obtained from a multicenter cross-sectional survey. We strategically selected five hospitals that provide ART services, which are geographically distributed across China, including Shanghai (municipality), Shandong (East China), Ningxia (West China), Guangdong (South China), and Hubei (Central China). A quota sampling method was used to recruit individuals with infertility undergoing ART treatment. The inclusion criteria were as follows: (i) individuals diagnosed with infertility based on the WHO definition of “a disease of the male or female reproductive system defined by the failure to achieve a pregnancy after 12 months or more of regular unprotected sexual intercourse” (World Health Organization 2023a); (ii) individuals undergoing one of the five ARTs approved by the National Health Commission of China; (iii) individuals aged between 18 and 49 years; (iv) individuals with infertility undergoing embryo transfer or AI at the time of the survey, indicating that they were actively completing a full cycle of ART. After giving informed consent, participants were invited to complete the questionnaire while waiting outside the operating room for intrauterine insemination or embryo transfer procedures. Female respondents were selected as the primary respondents for the following reasons: (i) female factors account for the majority of infertility cases (Liang *et al*. 2025); (ii) ART procedures are primarily performed on the women’s bodies, even in cases of male infertility, making women generally more familiar with the treatment process and related costs (Webair *et al*. 2021); (iii) males are more likely to decline survey invitation due to stigma (Webair *et al*. 2021, Liang *et al*. 2025).

### Questionnaire

The contingent valuation method (CVM) is a widely used technique for measuring individuals’ stated preferences. Participants were asked to imagine themselves in hypothetical scenarios—such as experiencing a specific disease—and then to indicate their WTP for a service or good that would improve their well-being. The CVM has been widely adopted by researchers worldwide, particularly in WTP (Klose 1999). Moreover, CVM has demonstrated robust validation in estimating WTP for ARTs across different countries (Settumba *et al*. 2019, Fenwick *et al*. 2023). The scenarios in this study were developed based on the guidelines for contingent valuation (Carson 2000).

The contingent valuation investigation of WTP in our study included three scenarios: AI, IVF, and PGT. Each scenario was designed based on the real-world efficacy and cost of these technologies in mainland China, as detailed in Supplementary S1. Since all respondents in this study were individuals with infertility, it was unnecessary to hypothesize that participants themselves had infertility; thus, this aspect was excluded from the scenario descriptions. Each scenario presented participants with information on pregnancy rates, treatment complexity, and related costs for each technology.

To assess WTP for ARTs, we used a close-ended dichotomous choice format followed by a payment card technique. Participants were first asked whether they were willing to pay a specified initial bid amount. Those who responded “yes” were presented with a set of higher bid ranges, while those who responded “no” were shown lower bid ranges. Participants then selected the range that best reflected their WTP for the treatment. In Scenario A, participants were asked to state their WTP for IVF, with an initial bid of RMB 40 000. In Scenario B, the initial bid for AI was RMB 7000. In Scenario C, the initial bid for PGT was RMB 60 000. The initial, minimum, and maximum bid values for each scenario were determined based on the cost per cycle reported in previous studies (Bai *et al*. 2018, Qiao *et al*. 2021, Zhang and Yi 2021, Wang *et al*. 2022). The detailed bidding questions for each scenario are presented in Supplementary S2.

In addition to the CVM, the questionnaire also collected information on age, sex, socioeconomic status, and disease-related details. According to previous empirical studies and reviews, these factors may influence WTP for ART among individuals with infertility (Poder *et al*. 2014, Dieng *et al*. 2020, Fenwick *et al*. 2023). Specifically, socioeconomic status included educational attainment (1 = middle school and below; 2 = high school; 3 = junior college; 4 = university and above), enrollment in basic and supplementary health insurance, employment status (1 = employed; 2 = non-employed; 3 = other), and economic status. Basic health insurance schemes in China includes the Urban-Rural Residents’ Basic Medical Insurance (URRBMI) and Urban Employees’ Basic Medical Insurance (UEBMI). Enrollment in supplementary health insurance was coded as a dichotomous variable, (0 = No; 1 = Yes). The economic status of the respondents was measured by annual household income per capita in the previous year.

Disease-related variables included the cause of infertility (classified as male factor, female factor, combined factor, unexplained infertility, or other), the occurrence of complications during infertility treatment, and the presence of any chronic diseases. Female factors included tubal factors, diminished ovarian reserve, ovulatory dysfunction, and endometriosis. Male factors included oligoasthenospermia, teratospermia, and azoospermia. The occurrence of complications was a binary variable (No/Yes) indicating whether the respondent had experienced any of the following conditions during ART treatment: (i) moderate to severe ovarian hyperstimulation syndrome, (ii) postoperative bleeding (>300 ml), (iii) postoperative pelvic infection, or (iv) other clinically reported complications. Patient-physician communication about costs was measured using a four-category nominal variable (Rai *et al*. 2020), based on responses to the question: “Has your physician ever discussed the total medical cost of ART with you?” The responses were categorized into four levels: (i) never discussed, (ii) briefly discussed, (iii) discussed in detail, (iv) I do not remember.

Prior to the pilot survey, the content of the questionnaire was validated by several experts to ensure the relevance and clarity of each question. Expert consultation was conducted using a focus group discussion approach. Experts were purposively selected to provide multidisciplinary suggestions, including: (i) nurses familiar with the clinical procedures of ART; (ii) financial personnel with expertise in the cost of ART services; (iii) health economists experienced in using CVM to assess WTP; (iv) researchers specializing in infertility-related health policy. Three rounds of focus group discussion were held on 28 June, 3 July, and 5 July 2023. During these sessions, experts reviewed each item in the questionnaire, provided comments on content accuracy and clarity, and suggested revisions. We reviewed and discussed the feedback, and all modifications were finalized through group consensus to improve the questionnaire’s validity and feasibility.

Before the formal survey, a pilot survey was conducted in Shandong province with 45 individuals with infertility. Based on the participants’ feedback, we verified the comprehensibility of the questionnaire and the appropriateness of the bidding values. As no substantial revisions were required, the questionnaire remained unchanged. The questionnaire was completed by one member of an infertile couple via a web-based survey platform (Wenjuanxing) and checked by trained interviewers before submission. The formal survey was conducted between August and November 2023.

### Statistical analysis

Descriptive statistics were used to summarize respondents’ characteristics, including the number and proportions of participants in each group. Responses from the CVM were converted into interval-censored data. For instance, if a respondent selected a range (e.g. “RMB 5000–5999”), the lower bound (wtp_lb) was set to 5000, and the upper bound (wtp_ub) to 5999; For responses indicating a value “less than RMB 5000” or “more than RMB 10000,” the data were coded as left-censored (wtp_lb=., wtp_ub=4999) or right-censored (wtp_lb=10000, wtp_ub=.), respectively. Interval regression analysis—a well-established method for analyzing interval-dependent variables derived from CVM studies (Angell *et al*. 2018, Xu *et al*. 2024)—was performed to estimate WTP in each scenario and explore its potential correlations with demographic, socioeconomic and disease-related factors. The selection of explanatory variables was based on previous reviews and empirical studies (Steigenberger *et al*. 2022, Fenwick *et al*. 2023, Keller *et al*. 2023). Mean WTP and 95% confidence intervals (CIs) were expressed in RMB, with conversions to 2023 US dollars using exchange rate ($1 = RMB 7.08). Additionally, WTP was expressed as a proportion of China’s 2023 GDP per capita (RMB 89 424) (National Bureau of Statistics of China 2024). Mean WTP and its associated factors were estimated using maximum likelihood methods with the “intreg” command and robust errors in STATA 15.0 (StataCorp 2017). A two-tailed *P*-value of .05 was considered statistically significant. All data analyses were performed using Stata 15.0 (StataCorp 2017).

This study obtained ethics approval from the Ethics Committee of the Center for Health Management and Policy Research, of the authors’ institute. All respondents provided informed consent before participating in the survey.

## Results

### Basic characteristics of respondents

The data collection process was conducted as planned. The response rate was ∼80%, with the main reasons for refusal to participate being lack of time and the stigma associated with infertility. A detailed overview of the participants’ demographic and socio-economic characteristics is presented in Table 1. A total of 570 respondents completed the formal survey, with a mean age of 33.0 years and a predominance of female participants (94.4%). Nearly a quarter of the participants had an educational level of middle school or below (26.1%), while more than one-third had attained a university education. Most respondents were covered by basic health insurance (97.2%), although the majority did not enroll in supplementary health insurance (91.9%). With regard to the cause of infertility, female factors (37.5%) were more commonly reported than male factors (22.8%) or unexplained factors (23.0%). Geographically, the largest proportion of respondents were from Shandong (37.7%), while the smallest proportion were from Ningxia (9.8%).

**Table 1.** Sociodemographic and disease characteristics of study participants.

Variables	Categories	*N*	%
Total		570	100
Sex	Male	32	5.61
	Female	538	94.39
Age (mean, SD)		33.04	4.72
Education	Middle school and below	149	26.14
	High school	101	17.72
	Junior college	109	19.12
	University and above	211	37.02
Basic health insurance	No	16	2.81
	URRBMI	264	46.32
	UEBMI	290	50.88
Supplementary health insurance	No	524	91.93
	Yes	46	8.07
Employment	Employed	345	60.53
	Unemployed	66	11.58
	Non-employed	123	21.58
	Other	36	6.32
Household income per capita (mean, SD)		44 381.65	79 472.32
Causes	Female factor	214	37.54
	Male factor	130	22.81
	Combined factor	61	10.70
	Unexplained infertility	131	22.98
	Other	34	5.96
Chronic disease	No	456	80.00
	Yes	114	20.00
Complication	No	490	85.96
	Yes	80	14.04
Cost communication	None	121	21.23
	Brief	310	54.39
	Detailed	84	14.74
	Forget	55	9.65
Location	Shandong	215	37.72
	Shanghai	97	17.02
	Ningxia	56	9.82
	Guangdong	102	17.89
	Hubei	100	17.54

Supplementary health insurance includes private medical insurance, government medical insurance, medical assistance programs, and other employer-based supplementary insurance schemes. SD, standard deviation; URRBMI, Urban and Rural Residents’ Basic Medical Insurance; UEBMI, Urban Employees’ Basic Medical Insurance.

### WTP for ART

The predicted WTP is shown in Table 2. The mean WTP for one IVF cycle in Scenario A was RMB 30 163 (US $4259, 95% CI: RMB 29 650–30 675), equivalent to 0.34 times the GDP per capita. For one AI cycle in Scenario B, the predicted WTP was RMB 6046 (US $854, 95% CI: RMB 5987–6106), equivalent to 0.07 times the GDP per capita. Notably, the highest WTP among all technologies was observed for PGT in Scenario C, with an average WTP of RMB 47 234 (US $6669, 95% CI: RMB 46 435–48 033), equivalent to 0.53 times the GDP per capita.

**Table 2.** WTP for ARTs based on pregnancy rate among study participants.

ART	Mean (RMB)	Mean (US dollar)	95% CI (RMB)	Times of GDP per capita^a^
IVF	30 163	4259	(29 650, 30 675)	0.34
AI	6046	854	(5987, 6106)	0.07
PGT	47 234	6669	(46 435, 48 033)	0.53

ART, assisted reproductive technologies; IVF, *in vitro* fertilization; AI, artificial insemination; PGT, preimplantation genetic testing; RMB, renminbi; US dollar, 2023 US dollars converted using exchange rate; 95% CI, 95% confidence interval. ^a^Gross domestic product per capita in China 2023.

### Factors associated with WTP

Table 3 presents the results of interval regression analysis examining factors associated with WTP for ART. Notably, respondents’ age was negatively associated with WTP across all scenarios (*P* < .05), indicating that older individuals with infertility tended to report lower WTP for reproductive treatment. The relationship between education level and WTP was significant only for PGT. Respondents with a university degree or higher were willing to pay RMB 6609 more than those with a middle school education or lower (*P* < .05). The cause of infertility also showed a significant correlation with WTP. For IVF and PGT, respondents with male-factor or unexplained infertility reported lower WTP than those with female-factor infertility. Finally, cost communication with physicians was positively associated with patients’ WTP: compared with no communication, those reporting communication about cost had increased WTP of RMB 613 for AI and RMB 8814 for PGT, respectively.

**Table 3.** Factors associated with WTP for different ARTs: interval regression results.

Variable	Category	IVF^a^	AI^a^	PGT^a^
Sex	Male (reference group)			
	Female	−1814.57	−396.36	−11 706.77*
Age	(years)	−527.17**	−54.62*	−773.92***
Education	Middle school and below (reference group)			
	High school	474.40	−85.94	4046.13
	Junior college	700.54	−148.40	3915.34
	University and above	2079.40	517.85	6609.23*
Supplementary health insurance	No (reference group)			
	Yes	2306.80	−5.86	8059.95
Income per capita	(RMB)	0.02	0.002	0.03
Causes	Female factor (reference group)			
	Male factor	−7469.48**	286.54	−5948.31*
	Combined factor	2992.72	−291.06	6108.95
	Unexplained infertility	−7179.23**	9.92	−10 806.19***
	Other	−7153	−528.14	−7801.42
Chronic disease	No (reference group)			
	Yes	−1236.85	400.36	6552.71*
Complication	No (reference group)			
	Yes	−883.50	−115.65	302.11
Employment	Employed (reference group)			
	Unemployed	−735.40	84.46	525.22
	Non-employed	−3367.17	−259.7	−2182.73
	Other	1110.80	106.83	762.71
Cost communication	None (reference group)			
	Brief	2230.36	613.23*	8813.95**
	Detailed	2440.71	397.37	7163.19
	Forget	4437.16	571.51	9959.16*
Location	Shandong (reference group)			
	Shanghai	8260.47**	623.27	8855.87**
	Ningxia	2251.65	1042.02**	3522.80
	Guangdong	4104.68	614.93	3805.28
	Hubei	837.50	−197.88	−3040.26
Constant		47 510.64***	7233.29***	71 969.01***
*N*		570	570	570
Log pseudolikelihood		−925.25	−966.99	−897.37
Wald *χ*^2^		52.13	46.03	88.98
*P* > *χ*^2^		.0005	.0030	<.0001
AIC		1900.51	1983.98	1844.75
BIC		2009.15	2092.62	1953.39

IVF, *in vitro* fertilization; AI, artificial insemination; PGT, preimplantation genetic testing; AIC, Akaike Information Criterion; BIC, Bayesian Information Criterion. ^a^Dependent variables captured from CVM were converted into interval-censored data. *P < 0.05. **P < 0.01. ***P < 0.001.

## Discussion

### Main findings

This study estimated the WTP for three ART techniques among Chinese individuals with infertility using the CVM, and examined the factors associated with WTP. We found that the WTP varied significantly across the three techniques, ranging from 0.07 to 0.53 times the GDP per capita. Furthermore, age, education level, diagnosis of infertility, and cost communication were significant determinants of WTP for ART.

The mean WTP for PGT and IVF was considerably higher than that for AI, reflecting a potential association between WTP and the success rate of each ART. This finding was consistent with previous studies (Settumba *et al*. 2019, Fenwick *et al*. 2023, Keller *et al*. 2023). Specifically, the WTP for one IVF cycle among Chinese individuals with infertility was equivalent to 0.34 times the GDP per capita. Several publications have reported the WTP for one IVF cycle in other countries, such as Australia (0.09 times the GDP per capita) (Ryan 1996), UK (0.22–0.96 times the GDP per capita) (Ryan 1997, 1998, 2004, Abdulrahim *et al*. 2021, Skedgel *et al*. 2022), Israel (0.08–0.15 times the GDP per capita) (Spiegel *et al*. 2013, Gonen 2016), Brazil (0.69–0.85 times the GDP per capita) (Lima *et al*. 2020), Spain (0.55 times the GDP per capita), and the USA (0.42 times the GDP per capita) (Skedgel *et al*. 2022). Generally, the WTP for IVF among patients with infertility ranged from 0.09 to 0.96 times the GDP per capita across different settings (see Table 4). As Fenwick summarized, the lack of a standard or consensus on the WTP for IVF is partly due to methodological heterogeneity and variation in the clinical outcome measures (e.g. pregnancy vs. live birth rate) (Fenwick *et al*. 2023).

**Table 4.** Comparison of WTP for IVF across countries/regions between 1996 and 2022.

Author (year)	Country	Sampled population	Outcome measures	Method	GDP per capita^a^	Mean WTP	Times of GDP per capita
Ryan (1996)	Australia	Women at Integrated Fertility Services and their male partners	WTP for per cycle of IVF	Payment card technique	AUD 29 025	AUD 2506	0.09
Ryan (1997)	UK	Men and women who had been through IVF treatment	WTP for a cycle of IVF	Closed-ended technique	GBP 16 349	GBP 5101	0.31
Ryan (1998)	UK	Individuals receiving IVF treatment	WTP for current or next IVF cycle	Closed-ended technique	GBP 17 072	GBP 6552	0.38
Ryan (2004)	UK	Users of the *in vitro* fertilization (IVF) service	WTP for a further cycle of IVF	Dichotomous choice	GBP 22 048	GBP 4893	0.22
Abdulrahim *et al*. (2021)	UK	IVF patients	WTP for IVF using fresh embryo transfer	Discrete choice experiment	GBP 34 077	GBP 32 733	0.96
Spiegel *et al*. (2013)	Israel	IVF patients	WTP for one IVF cycle	Direct hypothetical questions about ‘maximum WTP’	USD 36 941	USD 5482	0.15
Gonen (2016)	Israel	IVF patients	WTP for one cycle of IVF	Direct hypothetical open-ended questions	USD 37 690	USD 3117	0.08
Lima *et al*. (2020)	Brazil	Couples seeking treatment for conjugal infertility	WTP for one cycle of IVF	Payment scale mode	USD 6923	USD 5854	0.85
				Direct question	USD 6923	USD 4800	0.69
Skedgel *et al*. (2022)	Spain	Respondents with experience of infertility or ART	WTP for ART per cycle	Discrete choice experiment	EUR 28 180	EUR 13 615	0.55
	UK				GBP 37 421	GBP 10 831	0.29
	USA				USD 76 330	USD 31 773	0.42

IVF, *in vitro* fertilization; WTP, willingness to pay. ^a^Gross domestic product per capita in the publication year.

In our study, the WTP for IVF among individuals with infertility in mainland China was lower than that in Australia but higher than that in Brazil, Spain and the USA. Several potential reasons may account for this variation. First, differences in study design and methodology used to estimate WTP—CVMs and outcome measures—may influence the results (Fenwick *et al*. 2023). Second, IVF success rates and costs varied across countries and regions. A common trend is that WTP tends to increase with both higher success rates and greater treatment costs (Neumann and Johannesson 1994, Settumba *et al*. 2019, Abdulrahim *et al*. 2021). Third, respondents’ attitudes toward infertility may have important impacts on WTP. A previous multi-country survey showed that public support for ART in China was significantly higher than in many other countries (Skedgel *et al*. 2021). Fourth, the coverage or reimbursement of ART costs by public funds or health insurance schemes may also affect WTP. The costs of ART were fully reimbursed in Israel and the UK, partially reimbursed in Australia, Spain and the USA, and not reimbursed in China until 2023 (International Federation of Fertility Societies 2022).

Consistent with previous studies, our findings confirmed that patients’ age and education level are significantly associated with WTP. Generally, older individuals with infertility were willing to pay less for ART compared with younger patients (Fenwick *et al*. 2023), possibly due to the lower clinical pregnancy and live birth rates of ART associated with advanced maternal age (Vitagliano *et al*. 2023). Moreover, patients with a university education or higher degree were found to be more willing to pay for PGT than those with lower education levels, even after controlling for covariates such as income. Two potential explanations may account for this finding. First, individuals with infertility who have higher education levels usually have more health literacy and knowledge about PGT, which has been identified as an important determinant of WTP for health services (Steigenberger *et al*. 2022). Second, individuals with infertility with higher education usually exhibit positive attitudes toward the use of PGT (Steigenberger *et al*. 2022). Notably, although studies from other countries have reported a positive association between higher educational attainment and WTP for IVF (Fenwick *et al*. 2023), no significant differences were observed among educational groups in this study. This may be attributed to sample characteristics. Participants in this study were individuals with infertility undergoing embryo transplantation or AI. They had not only strong fertility desires but also **access** to IVF, suggesting that the service was financially affordable for them. Therefore, the individuals with infertility included in this study were likely wealthier than the general public in other studies. Furthermore, the income per capita of the sampled respondents in this study (RMB 44 381) exceeded that of the general Chinese population (RMB 39 218) in 2023 (National Bureau of Statistics of China 2024). This suggests that the participants in our sample may have a relatively greater ability to pay for ART.

Furthermore, we found the WTP for ART varied across different causes of infertility. Infertile couples with male factors or unexplained infertility reported lower WTP for IVF and PGT than those with female-related infertility. One possible explanation is that diagnostic testing and treatment services differ by the type of infertility diagnosis (Katz *et al*. 2011). Couples with male factor or unexplained infertility tend to use fewer diagnosis and treatment services, resulting in lower cost (Quaas and Dokras 2008, Carson and Kallen 2021). As a result, they may have lower perceived value and report lower WTP for IVF and PGT (Skwara 2023).

Finally, we found that patient-physician communication about cost was positively associated with WTP for AI and PGT. As suggested by previous studies, appropriate price communication could increase WTP by providing patients with sufficient information and knowledge about health services and making them recognize the differential value of given services (Nagle *et al*. 2010, Steigenberger *et al*. 2022). Prior knowledge and information may impact WTP by improving perceived benefit and decreasing perceived risk. In addition, cost communication may affect WTP through improving price transparency and increasing patients’ trust in physicians and treatment care (Roosen *et al*. 2015, Morsink 2024).

### Strengths and limitations

This study has several strengths compared with previous research. First, there are marked differences in success rates, costs, and patient comfort across different ART techniques. Our study estimated the WTP for specific ART techniques rather than for general infertility treatment, which provides more detailed information about the perceived value of infertility treatment. Second, to the best of our knowledge, despite the urgent and substantial need for research on the financing of infertility treatment services (Njagi *et al*. 2023), studies on WTP for ART in LMICs remain scarce (Fenwick *et al*. 2023). Our findings contribute new evidence to ART financing and have important implications for policymakers in formulating public funding mechanisms or expanding health insurance coverage for infertility services.

However, this study has several important limitations. First, since participants were recruited from five provinces, there may be selection bias. The findings should be interpreted with caution, as their generalizability may be limited. Future research is needed to confirm and validate these findings. Second, the CVM used in this study may be subject to starting point bias—a common limitation of this approach. Starting point bias occurs when the initial bid value presented to respondents influences their stated WTP rather than reflecting their true valuation. In this study, the bidding values were set based on the real-world costs and success rates of each ART procedure to improve realism and relevance. However, this approach may have inadvertently anchored respondents’ valuations and led to an upward bias in the estimated WTP. This limitation should be considered when interpreting the findings, especially in the context of policy implications or cost-effectiveness analysis. Future research could reduce starting point bias by randomizing initial bid values across participants. Third, all respondents in this study were individuals with infertility who were currently undergoing ART and thus likely had better knowledge about healthcare costs and higher ability to pay. However, this sampling approach excluded individuals with infertility who did not pursue ART due to financial barriers, potentially introducing an upward bias in the estimated WTP for ART. Therefore, the sample selection strategy may have overestimated the actual WTP. This represents an important limitation in the generalization of our findings. Based on the findings from our study, future research involving all patients with infertility is expected to provide more comprehensive WTP estimations and inform national policies for resource allocation for ART. Fourth, 95% of the participants in this study were female, failing to reflect the actual female proportion in the infertility population. However, according to Fenwick’s systematic review, no significant differences in WTP for ART were observed between male and female individuals with infertility (Fenwick *et al*. 2023). Thus, the uneven sex distribution is not likely to cause serious bias in the estimation of WTP for ART. Fifth, due to sample size limitations, we did not conduct subgroup analyses in this study. Future studies should recruit a more diverse sample of individuals with infertility, including those who have not initiated ART, to explore disparities in access, affordability, and WTP across different socioeconomic groups. Given declining national birth rates, and the associated social and economic challenges created by an aging population, there is likely an externality value associated with an assisted birth that is not captured in these WTP values. Such analyses are essential to inform equitable fertility care policies and health insurance benefit package design.

### Policy implications

These findings have several important policy implications. First, China is facing the dual challenges of a low fertility rate and a high prevalence of infertility (Wang *et al*. 2024). In response, the government has taken measures to build a fertility-friendly society, including the incorporation of ART into the national health insurance scheme and the regulation of prices for ART services (Global Times 2025). Although selecting infertile patients currently undergoing ART may lead to an overestimation of WTP for ART, the values obtained from patients in this study can be used in conjunction with data from the general public and other stakeholders to inform health care coverage decisions. Second, the finding that the WTP for ART among individuals with infertility was not sensitive to economic status suggests strong desires for having children. Setting a higher cost-effectiveness threshold for ART—relative to interventions for other chronic diseases—and raising the reimbursement ceiling in health insurance schemes may help meet healthcare needs and support fertility goals (Wang 2024). Third, patient-physician communication, including discussions about costs and the provision of more information to patients, has been found to influence patients’ treatment decisions and is therefore recommended as part of shared decision-making (Oshima Lee and Emanuel 2013, Politi *et al*. 2023).

## Conclusion

We found the WTP among individuals with infertility in China was equivalent to 0.34 times GDP per capita for IVF, 0.07 times for AI, and 0.53 times for PGT, respectively. This study helps bridge the evidence gap in WTP for ART in China. These findings indicate strong support for including ART in the benefit package to prevent families from incurring financial catastrophe when accessing fertility services. These findings also inform that the government should establish appropriate cost-effectiveness thresholds and reimbursement ceilings for ART to improve affordability. Furthermore, we confirm that age and education are factors influencing WTP and provide new insights into WTP’s determinants, such as causes of infertility and patient-physician communication about cost. Given the potential overestimation bias in WTP resulting from sampling patients currently undergoing ART, future studies should conduct surveys among the general public and other stakeholders to obtain a broader consensus on the WTP for ART.

## Supplementary Material

czaf045_Supplementary_Data

## Data Availability

The data and code used in this study are available from the corresponding author upon reasonable request.

## Reflexivity statement

The authors include one female and five male and span multiple levels of seniority and disciplines. The authors include four professors and experienced researchers, one PhD candidates and one master. The authors have expertise in several areas including health economics, epidemiology, assisted reproduction technology and contingent valuation method. Two authors have extensive public health experience in global settings. Four authors are from low- and middle-income countries and tow authors are currently based in high-income countries.

## References

[czaf045-B1] Abdulrahim B, Scotland G, Bhattacharya S et al Assessing couples’ preferences for fresh or frozen embryo transfer: a discrete choice experiment. Hum Reprod 2021;36:2891–903. 10.1093/humrep/deab207 34550368

[czaf045-B2] Angell B, Cullen P, Laba T et al What is the value of a driver licence? A contingent valuation study of Australian adults. Transp Res Part A Policy Pract 2018;108:25–34. 10.1016/j.tra.2017.12.010

[czaf045-B3] Bai F, Liu C, Fan Y. Strategies for infertility prevention and control: a brief review. Chin J Public Health. 2018;34:1303–5. 10.11847/zgggws1113313

[czaf045-B4] Bai F, Wang DY, Fan YJ et al Assisted reproductive technology service availability, efficacy and safety in mainland China: 2016. Hum Reprod 2020;35:446–52. 10.1093/humrep/dez245 32020190

[czaf045-B5] Carson RT . Contingent valuation: a user's guide. Environ Sci Technol 2000;34:1413–8. 10.1021/es990728j

[czaf045-B6] Carson SA, Kallen AN. Diagnosis and management of infertility: a review. JAMA 2021;326:65–76. 10.1001/jama.2021.4788 34228062 PMC9302705

[czaf045-B7] Chambers GM, Sullivan EA, Ishihara O et al The economic impact of assisted reproductive technology: a review of selected developed countries. Fertil Steril 2009;91:2281–94. 10.1016/j.fertnstert.2009.04.029 19481642

[czaf045-B8] Dieng A, He J, Poder TG. Web comparison of three contingent valuation techniques in women of childbearing age: the case of ovulation induction in Quebec. Interact J Med Res 2020;9:e13355. 10.2196/13355 32027310 PMC7055751

[czaf045-B9] Dyer SJ, Sherwood K, Mcintyre D et al Catastrophic payment for assisted reproduction techniques with conventional ovarian stimulation in the public health sector of South Africa: frequency and coping strategies. Hum Reprod 2013;28:2755–64. 10.1093/humrep/det290 23878180

[czaf045-B10] Facey KM, Hansen HP, Single ANV. Patient Involvement in Health Technology Assessment. Singapore: Springer, 2017.

[czaf045-B11] Fenwick E, Eze A, D'hooghe T et al The value of treatment for infertility: a systematic literature review of willingness-to-pay thresholds and approaches for determining the cost effectiveness of fertility therapies. Best Pract Res Clin Obstet Gynaecol 2023;89:102340. 10.1016/j.bpobgyn.2023.102340 37290265

[czaf045-B12] Global Times . *Assisted reproductive technology covered in health insurance in China’s 31 provinces, regions and Xinjiang production and construction corps*. 2025. https://www.globaltimes.cn/page/202501/1327094.shtml (2 April 2025, date last accessed).

[czaf045-B13] Gonen LD . Social and private benefits of assisted reproductive technology: a national survey-based evaluation in Israel. J Comp Eff Res 2016;5:49–63. 10.2217/cer.15.51 26698829

[czaf045-B14] International Federation of Fertility Societies . *International Federation of Fertility Societies’ Surveillance (IFFS) 2022: Global Trends in Reproductive Policy and Practice*. 2022. https://www.iffsreproduction.org/wp-content/uploads/2022/10/IFFS-Surveillance-2022-Published.pdf (28 January 2023, date last accessed).

[czaf045-B15] Katz P, Showstack J, Smith JF et al Costs of infertility treatment: results from an 18-month prospective cohort study. Fertil Steril 2011;95:915–21. 10.1016/j.fertnstert.2010.11.026 21130988 PMC3043157

[czaf045-B16] Keller E, Botha W, Chambers GM. What features of fertility treatment do patients value? Price elasticity and willingness-to-pay values from a discrete choice experiment. Appl Health Econ Health Policy 2023;21:91–107. 10.1007/s40258-022-00764-7 36171511 PMC9834167

[czaf045-B17] Klose T . The contingent valuation method in health care. Health Policy 1999;47:97–123. 10.1016/S0168-8510(99)00010-X 10538292

[czaf045-B18] Lai SF, Choi SN, Ho YB et al A questionnaire survey on patients’ willingness to pay with reference to the waiting time of public in-vitro fertilization treatment in Hong Kong. Eur J Obstet Gynecol Reprod Biol 2021;258:430–6. 10.1016/j.ejogrb.2021.01.026 33550218

[czaf045-B19] Liang Y, Huang J, Zhao Q et al Global, regional, and national prevalence and trends of infertility among individuals of reproductive age (15–49 years) from 1990 to 2021, with projections to 2040. Hum Reprod 2025;40:529–44. 10.1093/humrep/deae292 39752330

[czaf045-B20] Lima SB, Antoniassi MP, Zylbersztejn DS et al Willingness of infertile couples to pay for in vitro fertilization treatment in the integrated human reproduction section of the Escola Paulista de Medicina, São Paulo Federal University. Value Health Reg Issues 2020;23:55–60. 10.1016/j.vhri.2020.03.003 32841901

[czaf045-B21] Lin P, Cangelosi MJ, Lee DW et al Willingness to pay for diagnostic technologies: a review of the contingent valuation literature. Value Health 2013;16:797–805. 10.1016/j.jval.2013.04.005 23947973

[czaf045-B22] Morsink MR . *Understanding the Impact of Business transparency on consumer trust and buying intention*. 2024. https://purl.utwente.nl/essays/103433 (22 September 2024, date last accessed).

[czaf045-B23] Nagle TT, Hogan J, Zale J. Price and Value Communication Strategies to Influence Willingness-to-Pay, the Strategy and Tactics of Pricing. London: Routledge, 2010.

[czaf045-B24] National Bureau of Statistics of China . China Statistical Yearbook. Beijing: China Statistics Press, 2024.

[czaf045-B25] National Health Commission of the People's Republic of China . *Updated list of medical institutions approved to carry out assisted reproductive technology and set up human sperm banks*. 2025. https://www.nhc.gov.cn/fys/c100077/202411/4fb6730657cd4d91adf798bfa49132a0.shtml (28 July 2025, date last accessed).

[czaf045-B26] Neumann PJ, Johannesson M. The willingness to pay for in vitro fertilization: a pilot study using contingent valuation. Med Care 1994;32:686–99. 10.1097/00005650-199407000-00003 8028404

[czaf045-B27] Njagi P, Groot W, Arsenijevic J et al Financial costs of assisted reproductive technology for patients in low- and middle-income countries: a systematic review. Hum Reprod Open 2023;2023:hoad007. 10.1093/hropen/hoad007 36959890 PMC10029849

[czaf045-B28] Oshima Lee E, Emanuel EJ. Shared decision making to improve care and reduce costs. N Engl J Med 2013;368:6–8. 10.1056/NEJMp1209500 23281971

[czaf045-B29] Poder TG, He J, Simard C et al Willingness to pay for ovulation induction treatment in case of WHO II anovulation: a study using the contingent valuation method. Patient Prefer Adherence 2014;8:1337–46. 10.2147/PPA.S67742 25328385 PMC4196787

[czaf045-B30] Politi MC, Housten AJ, Forcino RC et al Discussing cost and value in patient decision aids and shared decision making: a call to action. MDM Policy Pract 2023;8:23814683221148651. 10.1177/23814683221148651 36643615 PMC9834940

[czaf045-B31] Qiao J, Wang Y, Li X et al A Lancet Commission on 70 years of women's reproductive, maternal, newborn, child, and adolescent health in China. Lancet 2021;397:2497–536. 10.1016/S0140-6736(20)32708-2 34043953

[czaf045-B32] Quaas A, Dokras A. Diagnosis and treatment of unexplained infertility. Rev Obstet Gynecol. 2008;1:69–76. https://pmc.ncbi.nlm.nih.gov/articles/PMC2505167/18769664 PMC2505167

[czaf045-B33] Rai A, Zheng Z, Zhao J et al Patient–provider discussions about out-of-pocket costs of cancer care in the U.S. Am J Prev Med 2020;59:228–36. 10.1016/j.amepre.2020.02.017 32417019 PMC9278513

[czaf045-B34] Roosen J, Bieberstein A, Blanchemanche S et al Trust and willingness to pay for nanotechnology food. Food Policy 2015;52:75–83. 10.1016/j.foodpol.2014.12.004

[czaf045-B35] Ryan M . Using willingness to pay to assess the benefits of assisted reproductive techniques. Health Econ 1996;5:543–58. 10.1002/(SICI)1099-1050(199611)5:6<543::AID-HEC230>3.0.CO;2-R 9003941

[czaf045-B36] Ryan M . Should government fund assisted reproductive techniques? A study using willingness to pay. Appl Econ 1997;29:841–9. 10.1080/000368497326499

[czaf045-B37] Ryan M . Valuing psychological factors in the provision of assisted reproductive techniques using the economic instrument of willingness to pay. J Econ Psychol 1998;19:179–204. 10.1016/S0167-4870(98)00003-8

[czaf045-B38] Ryan M . A comparison of stated preference methods for estimating monetary values. Health Econ 2004;13:291–6. 10.1002/hec.818 14981653

[czaf045-B39] Seifer DB, Wantman E, Sparks AE et al National survey of the Society for Assisted Reproductive Technology membership regarding insurance coverage for assisted reproductive technologies. Fertil Steril 2018;110:1081–1088.e1. 10.1016/j.fertnstert.2018.07.016 30396552

[czaf045-B40] Settumba SN, Shanahan M, Botha W et al Reliability and validity of the contingent valuation method for estimating willingness to pay: a case of in vitro fertilisation. Appl Health Econ Health Policy 2019;17:103–10. 10.1007/s40258-018-0433-3 30315488

[czaf045-B41] Skedgel C, Ralphs E, Finn E et al Is the public supportive and willing to pay for a national assistive reproductive therapies programme? Results from a multicountry survey. BMJ Open 2021;11:e044986. 10.1136/bmjopen-2020-044986 PMC7949370 33692187

[czaf045-B42] Skedgel C, Ralphs E, Finn E et al How do people with experience of infertility value different aspects of assistive reproductive therapy? Results from a multi-country discrete choice experiment. Patient 2022;15:459–72. 10.1007/s40271-021-00563-7 34940935 PMC9197909

[czaf045-B43] Skwara F . Effects of mental accounting on purchase decision processes: a systematic review and research agenda. J Consum Behav 2023;22:1265–81. 10.1002/cb.2193

[czaf045-B44] Spiegel U, Gonen LD, Templeman J. Economic implications of in vitro fertilization using willingness to pay. J Public Health 2013;21:535–57. 10.1007/s10389-013-0582-7

[czaf045-B45] StataCorp . Stata Statistical Software: Release 15. College Station, TX: StataCorp LLC, 2017.

[czaf045-B46] Steigenberger C, Flatscher-Thoeni M, Siebert U et al Determinants of willingness to pay for health services: a systematic review of contingent valuation studies. Eur J Health Econ 2022;23:1455–82. 10.1007/s10198-022-01437-x 35166973 PMC8853086

[czaf045-B47] Vander Borght M, Wyns C. Fertility and infertility: definition and epidemiology. Clin Biochem 2018;62:2–10. 10.1016/j.clinbiochem.2018.03.012 29555319

[czaf045-B48] Vitagliano A, Paffoni A, Viganò P. Does maternal age affect assisted reproduction technology success rates after euploid embryo transfer? A systematic review and meta-analysis. Fertil Steril 2023;120:251–65. 10.1016/j.fertnstert.2023.02.036 36878347

[czaf045-B49] Wang X . *Medical insurance to cover assisted fertility services*. 2024. https://www.chinadaily.com.cn/a/202409/11/WS66e0f270a3103711928a749d.html (20 September 2024, date last accessed).

[czaf045-B50] Wang Y, Kong F, Fu Y et al How can China tackle its declining fertility rate? BMJ 2024;386:e078635. 10.1136/bmj-2023-078635 39214553 PMC11359724

[czaf045-B51] Wang L, Zhu Y, Wang T et al Feasibility analysis of incorporating infertility into medical insurance in China. Front Endocrinol (Lausanne) 2022;13:967739. 10.3389/fendo.2022.967739 36133311 PMC9483096

[czaf045-B52] Webair HH, Ismail TAT, Ismail SB et al Patient-centered infertility questionnaire for female clients (PCIQ-F): part I: questionnaire development. BMC Med Res Methodol 2021;21:188. 10.1186/s12874-021-01376-w 34544388 PMC8454158

[czaf045-B53] World Health Organization . *Infertility*. 2023a. https://www.who.int/news-room/fact-sheets/detail/infertility (19 February 2024, date last accessed).

[czaf045-B54] World Health Organization . *Infertility prevalence estimates, 1990–2021*. 2023b. https://www.who.int/publications/i/item/978920068315 (19 February 2024, date last accessed).

[czaf045-B55] Xu L, Chen M, Angell B et al Establishing cost-effectiveness threshold in China: a community survey of willingness to pay for a healthylife year. BMJ Glob Health 2024;9:e013070. 10.1136/bmjgh-2023-013070 PMC10806867 38195152

[czaf045-B56] Zhang X, Deng C, Huang X et al Annual report on assisted reproductive technology of Chinese Society of Reproductive Medicine in 2019. J Reprod Med 2022;31:1015–21. 10.3969/j.issn.1004-3845.2022.08.001

[czaf045-B57] Zhang Y, Yi Y. The First Country in Assisted Reproduction Technology. Beijing: China Economic Weekly, 2021.

[czaf045-B58] Zhou Z, Zheng D, Wu H et al Epidemiology of infertility in China: a population-based study. BJOG 2018;125:432–41. 10.1111/1471-0528.14966 29030908

