# Supplementary material for: Willingness to pay for assisted reproductive technologies among individuals with infertility in China
Source: Health Policy Plan. 2025 Jul 17;40(8):867–75. doi: 10.1093/heapol/czaf045 (PMC12448910; doi:10.1093/heapol/czaf045)
Supplement: czaf045_Supplementary_Data [file czaf045_supplementary_data.docx]

# Supplemental 1. Basic characteristics of assisted reproductive technologies in China

Table 1. Efficacy and cost of assisted reproductive technology in mainland China

| Technology | Clinical pregnancy rate (%) ^1^ | Delivery rate (%)^1^ | Cost per cycle |
| --- | --- | --- | --- |
| AI | 14.25–30.81 | 11.33–25.66 | 5000–8248^2, 3^ |
| IVF | 51.95 | 42.39 | 20000–80000^2-4^ |
| PGT | 63.27 | 54.08 | 30000–90000^5^ |

AI, artificial insemination; IVF, in vitro fertilization; PGT, preimplantation genetic testing.

If the infertility parents have a single gene disease and need embryo testing, it will cost at least 6000–7000 Yuan RMB more than the IVF.

# Supplemental 2. Contingent valuation survey question of assisted reproductive technology

**Hypothetical Scenario A: in vitro fertilization**

Now imagine that there is an assisted reproductive technology (ART) A, its clinical pregnancy rate per cycle is 50%, and the process is relatively complex and takes a lot of time and effort for you. You must pay this technology all by yourself. Considering your current economic situation:

**QA1**. Are you willing to pay RMB40,000 per cycle for this assisted reproductive technology?

No (Go to QA2) ⬜ Yes (Go to QA3) ⬜

**QA2**. You are not willing to pay RMB40,000 for one IVF cycle. Please indicate the maximum amount you would pay for one IVF cycle:

Less than 20,000 ⬜ 20,000-29,999 ⬜ 30,000-39,999 ⬜

**Go to Question QA4**

**QA3**. You are willing to pay RMB40,000 for one IVF cycle. Please indicate the maximum amount you would pay for one IVF cycle:

40,000-49,999 ⬜ 50,000-59,999 ⬜ 60,000-69,999 ⬜ More than 70,000 ⬜

**Hypothetical Scenario B: Artificial insemination**

Now imagine that there is an assisted reproductive technology (ART) B, its clinical pregnancy rate per cycle is 20%, the operation is simple and short, and the female does not have to endure the pain of surgery. You must pay this technology all by yourself. Considering your current economic situation:

**QB1**. Are you willing to pay RMB7,000 per cycle for this assisted reproductive technology?

No (Go to QB2) ⬜ Yes (Go to QB3) ⬜

**QB2**. You are not willing to pay RMB7,000 for one AI cycle. Please indicate the maximum amount you would pay for one IVF cycle:

Less than 5,000 ⬜ 5,000-5,999 ⬜ 6,000-6,999 ⬜

**Go to Question QB4**

**QB3**. You are willing to pay 7,000 Yuan RMB for one AI cycle. Please indicate the maximum amount you would pay for one AI cycle:

7,000-7,999 ⬜ 8,000-8,999 ⬜ 9,000-9,999 ⬜ More than 10,000 ⬜

**Hypothetical Scenario C: Preimplantation genetic testing**

Now imagine that there is an assisted reproductive technology (ART) C, its clinical pregnancy rate per cycle is 60%, and the process is relatively complex and takes a lot of time and effort for you. Its strength was it can reduce the risk of some genetic diseases. You must pay this technology all by yourself. Considering your current economic situation:

**QC1**. Are you willing to pay RMB60,000 per cycle for this assisted reproductive technology?

No (Go to QC2) ⬜ Yes (Go to QC3) ⬜

**QC2**. You are not willing to pay RMB60,000 for one PGT cycle. Please indicate the maximum amount you would pay for one PGT cycle:

Less than 40,000 ⬜ 40,000-49,999 ⬜ 50,000-59,999 ⬜

**Go to Question QC4**

**QC3**. You are willing to spend RMB60,000 for one PGT cycle. Please indicate the maximum amount you would pay for one PGT cycle:

60,00-69,999 ⬜ 70,000-79,999 ⬜ 80,000-89,999 ⬜ More than 90,000 ⬜

**References:**

**1.** Zhang X, Deng C, Huang X, et al. Annual report on assissted reproductive technology of Chinese Society of Reproductive Medicine in 2019. *J Reprod Med.* 2022;31(8):1015-1021.

**2.** Bai F, Liu C, Fan Y. Strategies for infertility prevention and control: a brief review. *Chin J Public Health.* 2018;34(9):1303-1305.

**3.** Zhang Y, Yi Y. The first country in assisted reproduction technology. *China Economic Weekly.* 2021-6-15, 2021.

**4.** Qiao J, Wang Y, Li X, et al. A Lancet Commission on 70 years of women's reproductive, maternal, newborn, child, and adolescent health in China. *Lancet.* 2021;397(10293):2497-2536.

**5.** Wang L, Zhu Y, Wang T, et al. Feasibility analysis of incorporating infertility into medical insurance in China. *Front Endocrinol (Lausanne).* 2022;13.
